# Effects of fungal infection on feeding and survival of *Anopheles gambiae* (Diptera: Culicidae) on plant sugars

**DOI:** 10.1186/s13071-015-0654-3

**Published:** 2015-01-20

**Authors:** Sopher N Ondiaka, Elizabeth W Masinde, Constantianus JM Koenraadt, Willem Takken, Wolfgang R Mukabana

**Affiliations:** International Centre of Insect Physiology and Ecology, P.O. Box 30772 GPO, Nairobi, Kenya; Laboratory of Entomology, Wageningen University and Research Centre, P.O. Box 8031, Wageningen, EH 6700 The Netherlands; School of Biological Sciences, University of Nairobi, P.O. Box 30197 GPO, Nairobi, Kenya

**Keywords:** *Metarhizium anisopliae*, *Anopheles gambiae s.s*, Host plants, Sugar feeding, Malaria vector

## Abstract

**Background:**

The entomopathogenic fungus *Metarhizium anisopliae* shows great promise for the control of adult malaria vectors. A promising strategy for infection of mosquitoes is supplying the fungus at plant feeding sites.

**Methods:**

We evaluated the survival of fungus-exposed *Anopheles gambiae* mosquitoes (males and females) fed on 6% glucose and on sugars of *Ricinus communis* (Castor oil plant) and *Parthenium hysterophorus* (Santa Maria feverfew weed). Further, we determined the feeding propensity, quantity of sugar ingested and its digestion rate in the mosquitoes when fed on *R. communis* for 12 hours, one and three days post-exposure to fungus. The anthrone test was employed to detect the presence of sugar in each mosquito from which the quantity consumed and the digestion rates were estimated.

**Results:**

Fungus-exposed mosquitoes lived for significantly shorter periods than uninfected mosquitoes when both were fed on 6% glucose (7 versus 37 days), *R. communis* (7 versus 18 days) and *P. hysterophorus* (5 versus 7 days). Significantly fewer male and female mosquitoes, one and three days post-exposure to fungus, fed on *R. communis* compared to uninfected controls. Although the quantity of sugar ingested was similar between the treatment groups, fewer fungus-exposed than control mosquitoes ingested small, medium and large meals. Digestion rate was significantly slower in females one day after exposure to *M. anisopliae* compared to controls but remained the same in males. No change in digestion rate between treatments was observed three days after exposure.

**Conclusions:**

These results demonstrate that (a) entomopathogenic fungi strongly impact survival and sugar-feeding propensity of both sexes of the malaria vector *An. gambiae* but do not affect their potential to feed and digest meals, and (b) that plant sugar sources can be targeted as fungal delivery substrates. In addition, targeting males for population reduction using entomopathogenic fungi opens up a new strategy for mosquito vector control.

## Background

Studies have shown that fungal pathogens reduce survival of *Anopheles* mosquitoes to a level that prevents transmission of malaria parasites [1-3]. The fungi achieve this by reducing mosquito blood feeding [4,5] and fecundity [5]. Plant sugar acquired from floral and extrafloral nectaries, honeydew, damaged fruits and leaves is essential for mosquito survival [6-8]. It is the only nutritional source of adult males and a dietary supplement to blood for females. Sugar feeding is an early priority for both sexes, which typically have limited energy reserves upon emergence [9-14]. Besides survival and building of energy reserves, sugar enhances maturation of ovarian follicles in females and reproductive fitness in males [15].

Survival of the malaria mosquito *Anopheles gambiae* is assured with frequent feeding and ingestion of sizeable amounts of sugar meals [16] or by ingestion of small amounts of sugar at a time [6,8]. Recent studies have shown that mosquitoes feed on a wide variety of plants common in their natural habitats [17-20] with males preferentially feeding on the most rewarding sugar sources [21]. Sugars from some of these plants promote longer survival of both sexes, which enhances the vectorial capacity of females [16,22] and fitness and reproductive capacity of the males [21]. Most studies, though, have targeted females due to their significant role in malaria transmission with the contribution of males in the whole process often overlooked. As sugar feeding is central in the biology of adult mosquitoes, it is imperative to assess whether infection of mosquitoes with entomopathogenic fungi impacts sugar feeding.

This study investigated three aspects of plant sugar feeding behaviour of adult male and female *An. gambiae* mosquitoes under natural climatic conditions. These included (a) determining the survival of fungus-exposed *An. gambiae* mosquitoes when fed on glucose or plant sugars, (b) establishing the feeding propensity and the quantity of sugar ingested from plants by the infected mosquitoes and (c) assessing the digestion rate of sugar imbibed by fungus-exposed mosquitoes.

## Methods

### Mosquitoes

Experiments were carried out using laboratory-reared *Anopheles gambiae* Giles *sensu stricto* (hereafter termed *An. gambiae*) mosquitoes obtained from a colony established from wild gravid females collected at Mbita Point (000 25’S, 340 13’E), western Kenya in 1999. All mosquito life stages were maintained under ambient conditions in a mosquito insectary present at the Thomas Odhiambo Campus (TOC) of the International Centre of Insect Physiology and Ecology (*icipe*) located near Mbita Point Township in western Kenya. Larval and adult stages of the mosquitoes were raised using procedures described previously [23]. Both sexes were separated at emergence and held under ambient conditions in 30 × 30 × 30 cm cages inside a screenhouse. Before experiments, the insects were maintained either on an aqueous 6% glucose solution presented on filter paper wicks or on stem cuttings of *Ricinus communis* (Castor oil plant) and *Parthenium hysterophorus* (Santa Maria feverfew weed).

### Detection of plant sugars in mosquitoes using the anthrone test

The anthrone test was used to determine the presence of sugars in the mosquitoes. To do so standard sucrose solutions of different strengths in the series 1, 2, 4, 8, 16, 32, 64, 128 and 256 μg/μl were prepared. Initially, 25.6 g of reagent grade sucrose was dissolved in 50 ml of distilled water. More water was added gradually while mixing to make a 100 ml solution from which the serial dilutions were prepared. Distilled water served as the neutral liquid. These solutions were stored at −4°C. Diluted sulphuric acid was then prepared by mixing 380 ml concentrated sulphuric acid with 150 ml distilled water in a fume hood. The whole solution was cooled for 5 hours at room temperature and further for 12 hours in the refrigerator at 5°C before use. Anthrone reagent was then prepared by mixing 0.15 g of anthrone powder per 100 ml of the diluted sulphuric acid.

Two test tube racks were used with each rack holding one hundred 5-ml test tubes. A third rack was used to hold 10 test tubes for the standard sucrose solutions. The standards were prepared by pipetting 1 μl from each of the nine standard sucrose solutions into the nine separate test tubes. The tenth tube contained 1 μl of distilled water. The other two racks were used to hold both sexes of uninfected and *M. anisopliae*-exposed *An. gambiae* mosquitoes. Each tube held one mosquito. One drop each of chloroform and methanol in the ratio 1:1 was added to each tube containing mosquitoes to dissolve the cuticle. The racks were held in a fume hood where 0.5 ml of anthrone reagent was added to the standards and the tubes containing mosquitoes. The racks were then transferred into a water bath at room temperature for one hr. In the presence of sugar, the colour of the solutions changed from green to green-blue and further dark-blue depending on the amount of sugar. In absence of sugar, the colour of the sample was transparent yellow. After one hour the results were read by comparing the colour change in tubes containing mosquitoes and those with standard solutions.

### Fungal isolate

The entomopathogenic fungus *Metarhizium anisopliae* isolate ICIPE 30 was used (courtesy Dr. N.K. Maniania). This fungus was originally isolated from the stem borer *Busseola fusca* (Lepidoptera, Noctuidea) in Kendu Bay, western Kenya, in 1999 and has been maintained at the *icipe’s* Germplasm Centre. Conidia were produced on long rice as substrate [24]. Harvested spores were dried for 48 hours in a desiccator containing active silica gel and stored in a refrigerator (4-6°C) until required. The viability of spores was determined before being used in the experiments. Germination rates >85% after 24 hours on Sabouraud dextrose agar was considered adequate for use in the experiments.

### Fungal infection process

Transparent plastic cylinders (9 cm diameter; 15 cm height) were used to inoculate *An. gambiae* mosquitoes with spores of *M. anisopliae*. The inside vertical surface and the circular base of each cylinder were lined with white rough-surfaced velvex tissue papers that measured 28.6 × 14.3 cm (for vertical surface) and 9 cm in diameter (for circular base area). A piece of mosquito netting material was secured over the mouth of each cylinder using a rubber band. A hole was punched at the centre of the net to serve as an introduction point for experimental mosquitoes. Each cylinder was held in a slanting position and 0.1 g (approx. 1.0 × 10^11^ conidia/m^2^) of *M. anisopliae* spores were weighed and poured on the paper. Using both hands, the cylinders were rolled several times until the papers were uniformly covered by the spores. The inner and the base surfaces of the cylinder used for uninfected mosquitoes were lined with white rough paper without spores.

For survival experiments, a total of four cylinders with fungus and four cylinders without fungus were used. Of these, two cylinders with fungus and two cylinders without fungus were each used to infect male and female mosquitoes separately. Sixty 1-d-old female and male mosquitoes were introduced into each of their respective four cylinders. The insects were held for six hr being supplied with 6% glucose solution soaked in a cotton pad and placed on top of the netting material covering the cylinder. The mosquitoes were then transferred into four separate holding cages (30 × 30 × 30 cm) based on sex and treatment and were supplied with 6% glucose solution on filter paper wicks. The insects were maintained under ambient conditions inside a screenhouse at 28 ± 2°C and 70 ± 5% r.h. For studies to evaluate plant material this procedure was repeated to infect the mosquitoes but the insects were provided with floral parts of *R. communis* and *P. hysterophorous* separately as source of sugar instead of 6% glucose solution. The base of the floral parts was hooked on the netting material covering the mouth of each cylinder using a tooth pick to suspend the plant inside the cylinder.

For sugar quantity and digestion rate experiments, five cylinders with fungus and three cylinders without fungus were used to infect female mosquitoes. The same numbers of cylinders were used to infect male mosquitoes. The number of mosquitoes exposed to fungus was higher than for the uninfected group to adjust for mortality in the holding cages prior to the start of the experiments on days one and three post-exposure.

### Plant species

Two plant species namely *Ricinus communis* (Castor oil plant) and *Parthenium hysterophorus* (Santa Maria feverfew weed) were used. *Ricinus communis* has been demonstrated to enhance survival of *An. gambiae* and *P. hysterophorus* as most frequently visited by this mosquito species [19]. Five, fresh stems cut from each plant with leaves and floral parts intact were used in the study. The cuttings were collected from the agricultural field at *icipe*, Mbita Point, western Kenya where the plants grow naturally.

### Survival of *M. anisopliae*-infected *An. gambiae* mosquitoes fed on plant sugars

To study the effect of fungus on the survival of *M. anisopliae*-infected *An. gambiae* mosquitoes on plant sugars, one hundred male and 100 female *An. gambiae* mosquitoes exposed to *M. anisopliae* for six hours upon emergence were held in separate cages (30 × 30 × 30 cm). Each cage was supplied with 250-ml flat bottomed conical flask containing 200-ml filtered Lake Victoria water and five stems (with leaves and floral parts intact) of *R. communis*. The stems were replaced every two days. Mosquito mortality was recorded daily to determine the length of time over which the mosquitoes survived. Dead individuals were plated in a Petri dish lined with wet filter paper and incubated at 28 ± 2°C. Fungal growth on mosquito cadavers was observed after three days at 400× magnification under a compound microscope. The experiment was replicated four times. This procedure was repeated using *P. hysterophorus* as an alternative test plant and 6% glucose solution on filter paper wicks as a control. Glucose solution was changed every two days.

### Quantity of sugar imbibed by *M. anisopliae*-infected *An. gambiae* mosquitoes

In order to determine the quantity of sugar imbibed by *M. anisopliae*-infected *An. gambiae* mosquitoes, preliminary experiments were conducted to determine the length of time required for individual mosquitoes to feed fully. Three groups of female mosquitoes each composed of 50 individuals were fed on stems (with leaves and floral parts intact) of *R. communis* for separate periods of 6, 12 and 24 hours. The experiments were replicated four times over time. This procedure was repeated with male mosquitoes. Both male and female mosquitoes took 12 hours to satiate. Thus, the amount of sugar ingested from stems of *R. communis* by male and female mosquitoes, one and three days post-exposure to *M. anisopliae*, was evaluated after every 12 hours of feeding. One day after exposure to fungus, fifty male and female mosquitoes were aspirated, each from their respective uninfected and fungus-exposed cages and released into four separate cages. The insects were starved for 6 hrs prior to introduction of a 250-ml conical flask containing stems of *R. communis* in each cage. After 12 hours of feeding, the insects were removed from the cages and held in four separate collection cups. The insects were held inside a refrigerator at 4°C for 30 min and their sugar levels were quantified following the procedure earlier described on ‘detection of plant sugars in mosquitoes using anthrone test’. The experiment was replicated four times. This procedure was repeated with mosquitoes three days post-infection.

### Sugar digestion rate of *M. anisopliae*-infected *An. gambiae* mosquitoes

The digestion rate of *An. gambiae* mosquitoes exposed to *M. anisopliae* was determined by feeding males and females on *R. communis*. One day after exposure to fungus, 50 mosquitoes were aspirated from each cage holding uninfected and fungus-exposed mosquitoes and released into four separate cages. The mosquitoes were starved for 6 hours prior to introduction of the plant in a 250-ml conical flask in each cage. Mosquitoes were allowed to feed on *R. communis* for 12 hours after which the flasks containing the plant were removed from the cages. Fifty mosquitoes that appeared fully fed were also removed and held in separate cages from where ten mosquitoes were removed at an interval of 8 hours starting from time zero through to 32 hours post-feeding. Removed mosquitoes were held inside a refrigerator at 4°C for 30 min and the quantity of sugar in them, and by extension digestion rate, determined following the procedure earlier described. The experiment was replicated four times. The same procedure was repeated with groups of mosquitoes three days post-exposure to *M. anisopliae*.

### Ethical approval

Ethical approval for this study was given by the Kenya National Ethical Review Committee located at the Kenya Medical Research Institute (NON-SSC Protocol number 203).

### Statistics

Survival of uninfected and *M. anisopliae*-infected mosquitoes on glucose (6%), *R. communis* and *P. hysterophorous* was calculated by expressing the number of mosquitoes that succumbed to mortality as a percentage of the total number tested. Differences in survival between uninfected and fungus-infected groups were estimated using Cox regression analysis. Mortality rates, expressed as Hazard Ratio (HR) were used to estimate the risk of dying when infected compared to when not infected with fungus. To evaluate effects of infection on the amount of sugar ingested by infected (one and three days post-exposure) and control mosquitoes, first the number of mosquitoes that had fed on *R. communis* was expressed as a percentage of the total number tested. Further, the number of mosquitoes that imbibed small, medium and large quantities of sugar, respectively, was expressed as the mean percentage of the total number of mosquitoes tested. The difference between control and fungus-infected mosquitoes was calculated with the Chi square (*χ*^2^) test [25]. The digestion rate of the sugar ingested by mosquitoes one and three days post-exposure was each calculated by logistic regression. Logistic relationships for uninfected and fungus-exposed mosquitoes were fitted to describe sugar detection success for each time elapsed since feeding. The difference between uninfected and fungus-exposed mosquitoes was estimated by the Chi square (*χ*^2^) test. All analyses were conducted using SPSS (version 17.0)

## Results

### Survival of *M. anisopliae*-infected *An. gambiae* mosquitoes fed on plant sugars

**Figure 1:**
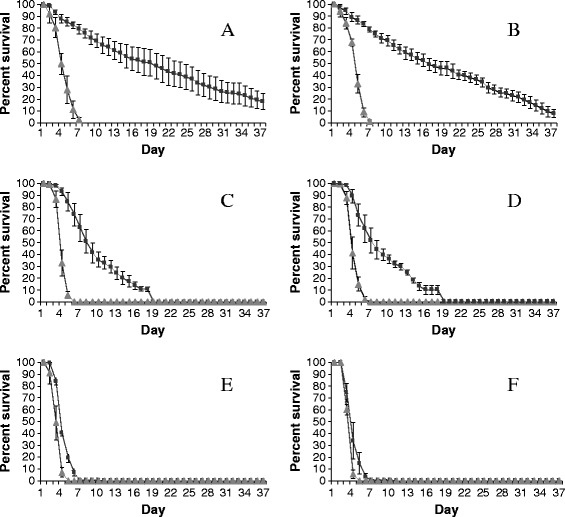
**Survival of uninfected and** ***M. anisopliae*** **-infected** ***An. gambiae*** **females (Panel A, C and E) and males (Panel B, D and F) when fed on: (i) 6%** **glucose (panel A and B); (ii)** ***Ricinus communis*** **(panel C and D) and (iii)** ***Parthenium hysterophorus*** **(Panel E and F).** Uninfected and *M. anisopliae*-infected mosquitoes are depicted by closed squares and closed triangles, respectively.

Infection with *M. anisopliae* reduced the survival of both sexes of *An. gambiae* with 100% mortality occurring within seven days compared to ≥ seven days with uninfected mosquitoes irrespective of the nutritional source (Figure 1). Survival of infected male and female mosquitoes in each nutritional group was significantly different from their respective controls. For example, the daily risk of death for both sexes was eight-fold greater on 6% glucose; four-fold (males) and eight-fold (females) greater on *R. communis* and two-fold greater for both sexes on *P. hysterophorus* relative to their controls (Table 1). In uninfected mosquitoes, the daily risk of death was three-fold greater for both males (HR = 3.4 [95% CI = 2.91 - 4.21], P = 0.0001) and females (HR = 2.9 [95% CI = 2.45 - 3.55], P = 0.0001) fed on *R. communis* and 14-fold greater for males (HR = 14.1 [95% CI = 11.33 - 17.6], P = 0.0001) and 13-fold greater for females (HR = 13.4 [95% CI = 10.71 - 16.8], P = 0.0001) fed on *P. hysterophorus* relative to 6% glucose. Therefore, *P. hysterophorus* caused a drastic reduction in the survival of mosquitoes regardless of fungal infection. Between sexes, survival rate over time in each nutritional regime was not different. Mycosis test results indicated high infection rates (>77%) in fungus-exposed male and female mosquitoes. No fungal conidia were observed on the cadavers of the control mosquitoes.

**Table 1:** **Survival analysis of** ***An. gambiae*** **mosquitoes infected with** ***M. anisopliae*** **and fed on different nutritional sources; data show Cox regression Hazard Ratio (HR) outcomes (95**% **CI), statistical p-values are relative to the relevant control (not exposed to fungus)**

**Nutritional sources**	**HR (95%** **CI)**			
	**Male mosquitoes**	**P-value**	**Female mosquitoes**	**P-value**
Glucose (6%)	8.53 (6.68 - 10.89)	0.0001	7.64 (5.99 - 9.75)	0.0001
*Ricinus communis*	4.33 (3.59 - 5.23)	0.0001	8.21 (6.49 - 10.37)	0.0001
*Parthenium hysterophorus*	1.62 (1.40 - 1.89)	0.0001	2.15 (1.85 - 2.50)	0.0001

### Quantity of sugar imbibed by *M. anisopliae*-infected *An. gambiae* mosquitoes

**Figure 2:**
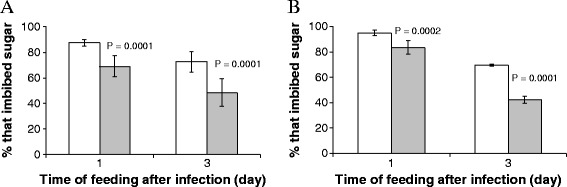
**Mean (± S.E) percentage of uninfected and** ***M. anisopliae*** **- infected** ***An. gambiae*** **males (Panel A) and females (Panel B) that imbibed sugar on exposure to** ***Ricinus communis*** **for 12 hr.** White and gray shaded bars represent uninfected and *M. anisopliae*-infected mosquitoes respectively. Level of statistical difference between treatments was calculated by Chi square **(** ***χ*** ^**2**^ **)** test. Each treatment tested 200 mosquitoes.

The quantity of sugar detected ranged from 1–64 μg. For easy analysis of the data, the mosquitoes were categorised as consumers of small, medium or large meals [26] if they imbibed 1–4 μg, 8–16 μg or 32–64 μg of sugar, respectively. Significantly fewer male and female mosquitoes exposed to fungus imbibed sugar from *R. communis* compared to mosquitoes not exposed to fungus (Figure 2). More uninfected than fungus-exposed mosquitoes ingested plant sugar. Fewer mosquitoes imbibed plant sugars at three days post-exposure than at one day post-exposure. Although fungus-exposed mosquitoes ingested less sugar than uninfected counterparts the differences were generally insignificant except for medium-feeding females three days post-exposure (Table 2) and small-feeding males, one and three days post-exposure (Table 3). Results from the mycosis test demonstrated high infection rates (>75%) in fungus-exposed males and females. No fungal hyphae were observed on the cadavers of control mosquitoes.

**Table 2:** **Mean (± S.E) percentage of uninfected and fungus-infected** ***An. gambiae*** **female mosquitoes (see Figure** 1 **) that imbibed different amounts of sugar when fed on** ***Ricinus communis*** **for 12 hours**

**Sugar quantity**	**Days post-exposure**	**N**	**Mean% (± S.E) of males that imbibed sugar**		***χ*** ^**2**^	**P**
			**Uninfected**	**Fungus-infected**		
Small	1	4	47.5 ± 8.54	38.0 ± 7.96	3.69	0.055
Medium		4	29.0 ± 4.51	26.0 ± 4.08	0.45	0.502
Large		4	18.5 ± 8.22	19.5 ± 6.29	0.07	0.799
Small	3	4	35.0 ± 4.12	30.0 ± 5.6	1.14	0.286
Medium		4	22.0 ± 2.22	6.0 ± 2.16	22.28	0.001
Large		4	12.0 ± 4.69	6.5 ± 6.5	3.60	0.058

One and three d post-exposure females were tested.

Statistical significance (P value) between the number of uninfected and fungus-infected mosquitoes in each category of sugar quantity imbibed was calculated by Chi square (***χ*** ^**2**^) test. Each treatment tested 200 mosquitoes.

**Table 3:** **Mean (± S.E) percentage of uninfected and fungus-infected** ***An. gambiae*** **male mosquitoes (see Figure** 1 **) that imbibed different amounts of sugar when fed on** ***Ricinus communis*** **for 12 hr**

**Sugar quantity**	**Days post-exposure**	**N**	**Mean% (± S.E) of males that imbibed sugar**		***χ*** ^**2**^	**P**
			**Uninfected**	**Fungus-infected**		
Small	1	4	56.0 ± 6.16	41.0 ± 9.47	9.01	0.003
Medium		4	22.0 ± 3.56	21.5 ± 4.19	0.02	0.903
Large		4	9.5 ± 1.71	6.5 ± 2.63	1.22	0.269
Small	3	4	52.0 ± 8.49	37.0 ± 6.14	9.11	0.003
Medium		4	16.5 ± 0.50	10.5 ± 7.37	3.08	0.079
Large		4	4.0 ± 2.45	1.0 ± 1.0	3.69	0.055

One and three d post-exposure males were tested.

Statistical significance (P value) between the number of uninfected and fungus-infected mosquitoes in each category of sugar quantity imbibed was calculated by Chi square (***χ*** ^**2**^) test. Each treatment tested 200 mosquitoes.

### Sugar digestion rate of *M. anisopliae*-infected *An. gambiae* mosquitoes

**Figure 3:**
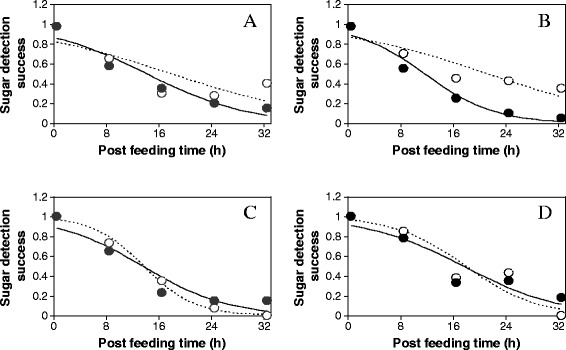
**Effect of infection with** ***M. anisopliae*** **on sugar detection success in** ***An. gambiae*** **mosquitoes.** Panels **A** and **B** represent sugar detection success in uninfected and in *M. anisopliae*-infected males and females respectively one day post-exposure with Panels **C** and **D** represent 3 d post-exposure when fed on *Ricinus communis* for 12 hr. Solid lines representing uninfected mosquitoes and dotted lines representing infected mosquitoes describe the fitted logistic relationships between sugar detection success for each time period since feeding: − Logit (sugar detection success) = ß_0_ + ß_1_ time. Circles denote observed values. Level of statistical difference between treatments was calculated by Chi square **(** ***χ*** ^**2**^ **)** test. Each treatment tested 200 mosquitoes.

The proportions of uninfected and *M. anisopliae*-infected mosquitoes testing positive for plant sugar consistently decreased over time (Figure 3). For each time period since feeding, more mosquitoes, one day post-exposure to fungus, tested positive for sugar than uninfected mosquitoes but the difference was not significant except in males at 32 hours (*χ*^2^ = 6.27; df = 1; P = 0.001) and in females at 24 hours (*χ*^2^ = 10.91; df = 1; P = 0.001) and 32 hours (*χ*^2^ = 11.25; df = 1; P = 0.001) of digestion. In addition, fewer mosquitoes, three days post-exposure, than controls tested positive for sugars until 16 hours in males and 24 hours in females after feeding with the differences significant at 32 hour of digestion (males: *χ*^2^ = 6.49; df = 1; P = 0.001; females *χ*^2^ = 7.67; df = 1; P = 0.006). Cumulative scores from time zero through to the 32 hour demonstrate that, more one day post-exposure males (52% versus 45%) and females (58% versus 39%) than controls tested positive for sugar. This was an overall indication that digestion rate was slower in fungus-exposed mosquitoes. The difference was only significant for females, one day post-exposure (Table 4). Further, the proportion of three day post-exposure males (43% versus 43%) and females (53% versus 53%) with sugar was equal to that of the controls. Hence, timing of fungal exposure only had an effect on sugar digestion in females. Results from mycosis tests indicated that, on average 73-81% of males and 78-85% of females were infected with fungus but no spores were observed on the cadaver of the control mosquitoes.

**Table 4:** **Proportion of uninfected and fungus-exposed** ***An. gambiae*** **mosquitoes that tested positive for sugar within 32 hr after feeding on** ***Ricinus communis*** **for 12 hr**

**Sex**	**Days post-exposure**	**N**	**% mosquitoes sugar positive**		***χ*** ^**2**^	**P**	**Percent (± S.E) infection**
			**Uninfected**	**Fungus-exposed**			
Male	1	4	45	52	1.96	0.161	73.0 ± 3.87 (146)
Female		4	38.5	58	15.29	0.001	78.0 ± 4.16 (156)
Male	3	4	43.5	43	0.01	0.920	81.0 ± 1.0 (162)
Female		4	52.5	53	0.01	0.920	85.0 ± 2.08 (170)

Males and females were tested one and three d post-exposure.

Statistical significance (P value) between the number of uninfected and fungus-infected mosquitoes in each category of sugar quantity imbibed was calculated by Chi square (***χ*** ^**2**^) test. Each treatment tested 200 mosquitoes.

## Discussion

Results of this study demonstrate that under ambient conditions, infection with the entomopathogenic fungus *M. anisopliae* reduced the daily survival of *An. gambiae* mosquitoes irrespective of the sugar source. Such a significant reduction in the survival of *An. gambiae* on glucose within 10 d after exposure to *M. anisopliae* has been reported previously under laboratory conditions [27-30]. Moreover, both *R. communis* and *P. hysterophorus* had a strong negative effect on survival of healthy mosquitoes. These findings are in agreement with other studies that reported longer survivorship of healthy *An. gambiae* mosquitoes fed on glucose than on plant-derived sugars [16,18,31].

Recent studies have shown that *An. gambiae* feed from a wide variety of plants and the quantity of sugar affects their survival [17,31]. Moreover, although sugar is present in the leaves, stems and floral parts of the plants, it is in the latter that different sugar types are highly concentrated [19]. Therefore, the lower survival on plant sugars relative to 6% glucose in this study may be due to the limited choice of nectar sources provided i.e. one plant choice instead of mixed plant choices and insufficient production of sugar by nectaries of the cut plants or accumulation of toxic substances due to interruption of nutrient circulation in the plant cuttings. The longer survival of uninfected mosquitoes on *R. communis* than *P. hysterophorus* may be attributed to the high amounts of digestible sugars produced [32] and therefore consumed in larger amounts from *R. communis* than *P. hysterophorus* [31]. The drastic reduction in the survival of mosquitoes on *P. hysterophorus* may be associated with the limited feeding resource points on the plant as reported in other studies where mosquitoes ingested sugars from the leaves only when compared to sugars imbibed from leaves, stems and floral parts in the case of *R. communis* plant [19]. Interestingly, the negative effects of plant sugars from *R. communis* and *P. hysterophorus* as reported may be entirely overcome when the insects are additionally offered a blood meal and these sugars then are highly beneficial by extending the survivorship [13,22,33,34].

Survivorship is a key feature that defines the vectorial capacity of malaria vectors [35,36]. Survival of *An. gambiae* mosquitoes on *R. communis* in this study was longer than the extrinsic incubation period of a pathogen that is as short as 10d for the malaria parasite *Plasmodium falciparum* [37-39]. This concurs with other studies on survival of *An. gambiae* on plant sugars [16,31]. As this occurred under semi-field conditions, it is likely that in field situations mosquitoes forage on a wide variety of plants to complete their dietary requirements, sustain longevity and with blood-supplement become efficient as malaria vectors. Therefore, reduction in the life-span of both sexes of *An. gambiae* by entomopathogenic fungi as demonstrated in this study could lead to a considerable reduction in malaria transmission.

Infection with fungi strongly reduced the proportion of mosquitoes that ingested sugar from *R. communis* independent of the time since infection. Interestingly, the feeding potential and the quantity of sugar assimilated by the mosquitoes that did feed remained similar between the treatment and the control groups. This is the first study to report on the impact of entomopathogenic fungi *M. anisopliae* on plant sugar feeding since reports to date about mosquitoes have addressed reduction of blood-feeding in fungus-infected females [1,2,5]. These findings corroborate our previous work that showed reduction in blood-feeding propensity in fungus-infected mosquitoes [40]. Both findings suggest that entomopathogenic fungi impose a similar effect on the feeding behaviour of mosquitoes irrespective of the food source. In other insect species, a significant reduction in feeding in the maize stem borer *Chilo partellus* (Swinhoe) larvae [41], adult thrips *Megalurothrips sjostedti* Trybom [42] and the variegated grasshopper, *Zonocerus variegatus* (Linnaeus) [43] occurred as early as one to four days after infection with the entomopathogenic fungus *M. anisopliae*. The normal feeding that we observed has also been reported in corn earworm, *Heliothis zea* (Boddie) larvae [44] infected with *B. bassiana*. The insects, however, die at a later stage, which may indicate that infection causes starvation due to physiological changes in infected hosts.

The reduction in sugar-feeding propensity may be attributed to three factors. First, infected mosquitoes may have fed as often as the uninfected ones but the sugar content was too low to be detected. Secondly, the sugar in infected mosquitoes may have already been digested and converted into a metabolic product which the anthrone test could not detect. Lastly, some of the ‘sick insects’ may have lost appetite [43], thus affecting their feeding ability [45-47]. The normal feeding in fungus-infected mosquitoes could be associated with dose [48] and the insect defense mechanism to fight the infection [49]. This is because the immune system of insects responds in defense of fungal attack as early as 12 h after exposure to the pathogen [50].

The study has further shown that infection by *M. anisopliae* has no effect on the digestion rate of sugar except in females, one day post-exposure. However, as the fungal infection progressed, fewer infected than uninfected mosquitoes (both sexes) tested sugar positive. Digestion of sugar in insects takes place in the crop and midgut and its rate is influenced by the meal size consumed, sugar concentration [51], metabolic rate [52] and the extent of energy reserves, among other factors. The mechanism that affects feeding rate due to pathogen attack may also affect the digestion process. Therefore, the slow digestion rate in early days of fungal infection is likely to be associated with the dose and the mechanical disruption of the midgut tissues by fungal toxins [46]. Furthermore, the increased breakdown of sugar as the infection advances could be associated with the need to replenish the teneral energy reserves depleted by invasive fungal pathogens in the insect haemolymph. These teneral reserves are critical for the survival of insects [13,53]. In the case where digestion rate between treatments was equal, it is likely that infected mosquitoes imbibed more sugar than controls for two purposes. First, to nourish the storage reserve this is the primary source of nourishment to the fungal pathogen [54]. Second, to replenish and store sugar in the crop for future use. This is because the accumulation of energy reserves retards digestion [6]. Between sexes, the proportion of individuals that tested positive to sugars did not differ in spite of their different synthesis of reserves. This concurs with what has been reported by van Handel [51].

The inability of fungus-exposed mosquitoes to sugar feed may pose some advantages. The life-span of both sexes could be reduced to less than five days. During this period, the mating ability of males may be compromised leading to fewer females getting inseminated. Although females can build their energy reserves from human blood, they may not survive long enough to become efficient malaria vectors. Therefore, if both sexes become infected early in life, this could lead to population suppression, incomplete development of the malaria parasite in females and reduction in malaria transmission [5,12]. Moreover, the ability of mosquitoes to feed on and digest sugars may negatively impact on the survival of both sexes and minimize human-mosquito contact. Thus, maintaining the normal rate of food consumption and digestion in fungus-infected insects for as long as possible benefits the fungal pathogen because this maximizes the amount of food available for the entomopathogen [33,55]. Further research however is needed to determine if a similar impact of fungus can occur in field situations.

The life of male mosquitoes is exclusively tied to the plant community. By focusing on fungal inoculation during plant feeding, therefore, both males and females are likely to become infected. Control strategies that target both sexes may lead to significant reduction in the prevalence and transmission of malaria and other mosquito borne diseases. In recent studies, the efficiency of plant attractants in attractive toxic sugar baits (ATSB) for the control of mosquitoes has been demonstrated [20,56-61]. The approach uses odour stationary traps baited with fermented ripe fruits and flower scent as attractants, a sugar solution as feeding stimulant and an oral pesticide [20]. The strategy can be adopted to infect and kill mosquitoes with entomopathogenic fungi during plant sugar feeding in two ways. Firstly, by spraying flowering plants with fungal conidia formulated in a suitable carrier that can withstand ultra-violent effects and retain spore virulence. This strategy however, requires assessment on the impact of fungal pathogens on non-target organisms especially pollinators as reported in a separate study with the use of ATSB [62]. Secondly, by spraying fungal conidia in traps baited with fruits and flowers and sugar solution or plant-derived synthetic odours to which mosquitoes respond to [63]. The use of plant odours in the traps will also increase the chance of infecting female mosquitoes harbouring malaria parasites with fungus since the ‘sick mosquitoes’ are reported to be highly attracted to sugar sources [64]. The first approach may be cost effective since preparation of attractants for the traps may be problematic. Also, more mosquitoes are likely to be targeted and killed by spraying the plants than by being attracted to the baited traps, as these are in competition with flowering plants. Nevertheless, research is needed to demonstrate the possibility of these proposed pathways and other unexplored approaches for infecting wild mosquitoes, particularly males, by entomopathogenic fungi.

## Conclusions

This study has demonstrated that infection with entomopathogenic fungi reduces survival and plant sugar-feeding ability of male and female *An. gambiae* mosquitoes, but not their potential to ingest and digest sugars, except in the late stages of fungal infection. By reducing survival, a fraction of the mosquito population is eliminated thus lowering the level of malaria transmission. The fact that infected mosquitoes continue to feed is an indication that they have a chance to sustain their physiological requirements including reproduction. This may delay mosquitoes from succumbing to infection quickly but may facilitate the occurrence of sub-lethal effects that can lead to reduction in fecundity and a further decline of mosquito population, hence disease transmission. The possibility of targeting male mosquitoes for population reduction by an entomopathogenic fungus opens a new strategy for mosquito vector control.
